# Supplementary material for: Tone classification of online medical services based on 1DCNN-BiLSTM
Source: PeerJ Comput Sci. 2024 Sep 23;10:e2325. doi: 10.7717/peerj-cs.2325 (PMC11623085; doi:10.7717/peerj-cs.2325)
Supplement: Article S1 [file peerj-cs-10-2325-s001.pdf]

Questionnaire on tone types of medical professional service providers

Thank you for participating in the Screening Survey on the Importance and Salience of Physician Tone. The data collected in this questionnaire will be used for scientific research and no personal privacy data will be collected, so please feel free to fill it out. There are no right or wrong answers to all questions, please fill them in based on your true thoughts.

1. Have you ever used online medical consultation services (such as "*Haodafu Online*", "*WeDoctor*", "*Chunyu Doctor*", "*Xunyiwenyao.com*", "*Dingxiang Doctor*" and other platforms)? [Single choice question] *

□Have used online consultation and received voice services used by doctors (such as doctors sending voice messages, making phone calls, and video chats)
Wait to communicate with me)

□I have used online consultation, but the doctor has not communicated with me in voice mode (for example, only uses text communication mode)

□I have never used online consultation, but I have been to offline hospitals for medical treatment

"Tone" refers to the way a person communicates through rhythmic changes in the voice, independent of the content of the conversation. Therefore, judge the doctor's tone based on your impression of the characteristics of their voice rather than the textual content of their words.

2. Based on the offline hospital consultation experience that I have participated in, what can I clearly feel about the doctor's tone? [Multiple choice questions] *

□Stressed (anxious)	□Angry
□Sympathy	□Steadiness
□Determination	□Dominant
□Tenderness	□Focus
□Genuine	□Satisfied
□Interested	

□Others (you can write more than one, please separate them with commas) ______________________________*

3. Based on the online consultation experience I have participated in, what can I clearly feel about the doctor's tone? [Multiple choice questions] *

□Stressed (anxious)	□Angry
□Sympathy	□Steadiness
□Determination	□Dominant
□Tenderness	□Focus

□Genuine □Satisfied
□Interested
□Others (you can write more than one, please separate them with commas)
______________________________*

4. Based on my experience of participating in offline hospital consultations, what kind of tone the doctor uses will seriously affect (increase or decrease) my satisfaction judgment with doctor services. [Multiple choice questions] *
□Stressed (anxious) □Angry
□Sympathy □Steadiness
□Determination □Dominant
□Tenderness □Focus
□Genuine □Satisfied
□Interested
□Others (you can write more than one, please separate them with commas)
______________________________*

5. Based on the online consultation experience I have participated in, the doctor's tone of voice will seriously affect (increase or decrease) my judgment of satisfaction with the doctor's service. [Multiple choice questions] *
□Stressed (anxious) □Angry
□Sympathy □Steadiness
□Determination □Dominant
□Tenderness □Focus
□Genuine □Satisfied
□Interested
□Others (you can write more than one, please separate them with commas)
______________________________*

6. Your gender [single choice question] *
□Male
□Female

7. Your age
[Enter a number from 10 (10 years old) to 70 (70 years old)] *
____

8. Your education level [single choice question] *
□Junior high school and below
□High school, technical secondary school
□College
□Undergraduate
□Master's degree and above

9. How tend to do you go to the doctor (online and offline combined) for consultation and consultation? [Single choice question] *

□Once a week □Once every half month

□Once a month □Once every two months

□Once every six months □Once a year

□More than once a year

10. According to your opinion, rate the following statement. The higher the score, the more you agree with it. A scale of 1 to 5 indicates from “strongly disagree” to “strongly agree”. [Matrix scale questions] *

	Strongly Disagree	Somewhat Disagree	Moderately Agree	Somewhat Agree	Strongly Agree
Doctors tend to authoritative	□	□	□	□	□
Doctors tend to professional	□	□	□	□	□
Doctors tend to warm	□	□	□	□	□
Doctors tend to be empathic	□	□	□	□	□
Doctors tend to be patient-centered	□	□	□	□	□
Doctors tend to only Focus on the disease itself	□	□	□	□	□
Doctors tend to trustworthy	□	□	□	□	□
Doctors tend to neglect to comfort me emotionally	□	□	□	□	□
