# Supplementary material for: Tone classification of online medical services based on 1DCNN-BiLSTM
Source: PeerJ Comput Sci. 2024 Sep 23;10:e2325. doi: 10.7717/peerj-cs.2325 (PMC11623085; doi:10.7717/peerj-cs.2325)
Supplement: Article S2 [file peerj-cs-10-2325-s002.pdf]

Audio labeling task

Thank you for participating in this labeling task. The task requires you to judge the tone of the doctor's voice in the audio recordings. This task will consist of 60 audio clips. After listening to each recording, please answer the related questions and fill in Table 1. There are no right or wrong answers. Please take note of the following:

1. Each audio clip has been processed for obfuscation. Please base your answers on the tone of the doctor's voice. Do not attempt to discern the content of the doctor's conversation. Your answers should be based on tone, not content, so you do not need to focus on what the doctor is saying;
2. Exiting midway or refreshing the page will not count towards task completion and will be considered as an invalid response;
3. Please listen to the audio clips and respond in a quiet environment;
4. The labeling time for each audio clip should not exceed 5 minutes;

Based on your feelings after listening to the voice, please rate the doctor's various tones in the audio (including "determination", "stress", "angry", "steadiness", "tenderness", "sympathy", "dominant", "focus" and "genuine"). The meaning of the tone is as follows:
Determination: The speaker sounds persistent, serious or serious;
Stress: The speaker sounds anxious and nervous;
Angry: The speaker sounds angry, angry or annoyed;
Steadiness: The speaker sounds calm, cautious or self-controlled;
Tenderness: The speaker sounds gentle;
Sympathy: The speaker sounds considerate, conveying concern;
Dominate: The speaker sounds like he or she is dominating the conversation, controlling or dominating the listener;
Focus: The speaker sounds focused on the task;
Genuine: The speaker sounds sincere;
The larger the score, the deeper the corresponding tone. Score meaning: "1" means "none", "2" means "rarely", "3" means "somewhat", "4" means "generally", "5" means "relative", "6" means "more", "7" means "very".

Score	None<br>1	Rarely<br>2	Somewhat<br>3	Generally<br>4	Relative<br>5	More<br>6	Very<br>7
Determination	□	□	□	□	□	□	□
Stress	□	□	□	□	□	□	□
Angry	□	□	□	□	□	□	□
Steadiness	□	□	□	□	□	□	□
Tenderness	□	□	□	□	□	□	□
Sympathy	□	□	□	□	□	□	□
Dominant	□	□	□	□	□	□	□
Focus	□	□	□	□	□	□	□
Genuine	□	□	□	□	□	□	□

Table 1 Scoring table for each voice
