# Supplementary material for: Tone classification of online medical services based on 1DCNN-BiLSTM
Source: PeerJ Comput Sci. 2024 Sep 23;10:e2325. doi: 10.7717/peerj-cs.2325 (PMC11623085; doi:10.7717/peerj-cs.2325)
Supplement: Article S3 [file peerj-cs-10-2325-s003.pdf]

医疗专业化服务者语气类型调查问卷

感谢您参与医生语气重要性和显著性的甄别调查。本问卷所采集数据用于科学研究，不收集个人隐私数据，请放心填写。所有问题没有对错之分，请您根据您的真实想法填写。

1. 您是否使用过在线医疗问诊服务（如好大夫在线、微医、春雨医生、寻医问药网和丁香园等平台等）？ [单选题] *

○使用过在线问诊，并且接受过医生采用的语音服务（如医生通过发送语音、打电话、视频聊天等与我交流）

○使用过在线问诊，但是医生没有采用过语音形式与我交流（如，只采用文本交流的模式）

○没有使用过在线问诊，但是我去过线下医院看病

“语气”是指声音的韵律变化所表现出的交流方式，独立于谈话内容。因此，请根据您印象中医生声音特征（而非话语文本内容）判断他们的语气。

2. 结合我参与过的线下医院问诊经历，我明显能感受到医生的哪些语气？ [多选题] *

□有压力（焦虑）的

□同情的

□坚定的

□温柔的

□真诚的

□感兴趣的

□愤怒的

□稳重的

□主导（支配）的

□专注的

□满足的

□其他（可写多个，请用顿号分隔）________________*

3. 结合我参与过的在线问诊经历，我明显能感受到医生的哪些语气？ [多选题] *

□有压力（焦虑）的

□同情的

□坚定的

□温柔的

□真诚的

□感兴趣的

□愤怒的

□稳重的

□主导（支配）的

□专注的

□满足的

□其他（可写多个，请用顿号分隔）________________*

4. 结合我参与过的线下医院问诊经历，医生什么样的语气会严重影响到（增加或减少）我对医生服务的满意度判断。 [多选题] *

□有压力（焦虑）的
□同情的
□坚定的
□温柔的
□真诚的
□感兴趣的
□愤怒的
□稳重的
□主导（支配）的
□专注的
□满足的
□其他（可写多个，请用顿号分隔）_________________*

5. 结合我参与过的在线问诊经历，医生什么样的语气会严重影响到（增加或减少）我对医生服务的满意度判断。 [多选题] *

□有压力（焦虑）的
□同情的
□坚定的
□温柔的
□真诚的
□感兴趣的
□愤怒的
□稳重的
□主导（支配）的
□专注的
□满足的
□其他（可写多个，请用顿号分隔）_________________*

6. 您的性别 [单选题] *

○男
○女

7. 您的年龄

[输入10(10 岁)到70(70 岁)的数字]*

____

8. 您的文化程度 [单选题] *

○初中及以下
○高中、中专
○大专
○本科
○硕士及以上

9. 您找（线上和线下合计）医生就诊、咨询的频次接近于？ [单选题] *

○每周一次
○每半月一次
○每月一次
○每两月一次
○每半年一次
○一年一次
○一年以上一次

10. 根据您的看法，对下面表述进行打分，分值越高表示越赞成。1~5 分依次表示从“非常不赞成”~“非常赞成”。[矩阵量表题] *

	非常不赞成	比较不赞成	一般赞成	比较赞成	非常赞成
医生往往是权威的	○	○	○	○	○
医生往往是专业的	○	○	○	○	○
医生往往是温暖的	○	○	○	○	○
医生往往是共情的	○	○	○	○	○
医生往往以病人为中心的	○	○	○	○	○
医生往往只关注于疾病本身	○	○	○	○	○
医生往往是值得信赖的	○	○	○	○	○
医生往往忽视对我情绪的安抚	○	○	○	○	○

再次感谢您的参与！
