# Supplementary material for: Tone classification of online medical services based on 1DCNN-BiLSTM
Source: PeerJ Comput Sci. 2024 Sep 23;10:e2325. doi: 10.7717/peerj-cs.2325 (PMC11623085; doi:10.7717/peerj-cs.2325)
Supplement: Article S4 [file peerj-cs-10-2325-s004.pdf]

Detailed description and calculation method of features

1. Zero-crossing rate

The zero-crossing rate (ZRC) is the rate of sign changes of the signal within a specific frame duration, or in other words, the number of times the signal crosses zero. ZRC is highly sensitive to "noise" in audio; when there is interference, the value of ZRC tends to be higher. Specifically, the calculation method for the zero-crossing rate of the ith frame of audio signal is as follows:

$$ZRC_i = \frac{1}{2L}\sum_{n}^{L} |\mathrm{sign}[x_i(n)] - \mathrm{sign}[x_i(n-1)]|$$

$$\mathrm{sign}[x(n)] = \begin{cases} 1, x(n) \geq 0 \\ -1, x(n) < 0 \end{cases}$$

Here, $x_i(n)$ represents the audio signal of the ith frame, and L represents the number of audio frames. $\frac{1}{2L}$ represents as a normalization factor to ensure that the ZRC value remains within a reasonable range.

2. Short-term energy

Short-term energy refers to the sum of squares of each frame signal, reflecting the strength of the signal's energy. It can have high explanatory power in the recognition of emotional activation states. The calculation method for the short-term energy of the ith frame of audio signal is as follows:

$$\mathrm{energy}_i = \frac{1}{L}\sum_{n=1}^{L} x_i^2(n)$$

Here, $x_i(n)$ represents the audio signal of the ith frame, L represents the number of samples in the audio frame.

3. Entropy of Energy

Energy entropy is calculated based on the normalized energy of multiple subframes in each audio frame. It can be interpreted as a measure of abrupt changes. The calculation method for the energy entropy of the ith frame of audio signal is as follows:

$$\mathrm{energy}_j = \frac{\mathrm{energy}_{\mathrm{subframe}\ j}}{\Sigma_k\ \mathrm{energy}_{\mathrm{subframe\ k}}}$$

$$H(i) = -\sum_{j=1}^{K} \text{energy}_j \log\left(\text{energy}_j\right)$$

Here, $energy_j$ represents the short-term energy of subframe j, $H(i)$ represents the energy entropy of the ith frame of the audio signal.

4. Spectral Centroid

The spectral centroid is the "center of gravity" of the spectrum. The higher the value of the spectral centroid, the more concentrated the energy of the signal is in higher frequencies. The spectral centroid of each audio frame can be calculated using the following equation:

$$X(k) = \text{DFT}[x(n)]_N = \sum_{n=0}^{N-1} x(n) \text{e}^{-j\frac{2\pi}{N}kn}$$

$$\text{spectral centroid}_i = \frac{\sum_{k=1}^{Wf_L} kX_i(k)}{\sum_{k=1}^{Wf_L} X_i(k)}$$

where $0 \leqslant K \leqslant N-1$, $X(k)$ represents the Fourier transform result of the kth frequency component, $x(n)$ represents the audio signal value at the nth sample point, N represents the total number of sample points, k represents the frequency component index, j represents the imaginary unit.

5. Spectral Spread

Spectral spread, also known as the second-order central moment of the spectrum, the spectral spread describes the distribution of the audio signal around the spectral centroid. The calculation formula is as follows:

$$\text{spectral spread}_i = \sqrt{\frac{\sum_{k=1}^{Wf_L} (k - c_i)^2 X_i(k)}{\sum_{k1}^{Wf_L} X_i(k)}}$$

Here, c_i represents spectral centroid of the ith frame.

6. Spectral Entropy

Spectral entropy is the entropy of normalized spectral energy for a group of sub-frames, characterizing the regularity of the power spectrum of a speech signal. The calculation formula is as follows:

$$\text{spectral entropy}_i = \frac{1}{\log N \left(\sum_{i=1}^{N} P(X_i(k))\log P(X_i(k))\right)}$$

Here, N represents the total number of subframes, $P(X_i(k))$ represents the power spectral density function of the kth frequency component of the ith frame.

7. Spectral Flux

Spectral flux captures the spectral change between two consecutive frames. It is calculated as the squared difference between the normalized magnitudes of spectra in two consecutive short-term windows. The calculation formula is as follows:

$$Fl_{(i,i-1)} = \sum_{k=1}^{Wf_L} (EN_i(k) - EN_{i-1}(k))^2$$

Here, $Fl_{(i,i-1)}$ represents the spectral flux between the ith frame and the i−1th frame. $EN_i(k)$ represents the normalized Discrete Fourier Transform (DFT) coefficient of the ith frame at frequency dimension k. The calculation formula is as follows:

$$EN_i(k) = \frac{X_i(k)}{\sum_{l=1}^{W_L f_L} X_i(l)}$$

8. Spectral Rolloff

Spectral rolloff represents the frequency below a specified percentage of the total spectral energy. In this paper, 90% is used as the standard. The calculation method for spectral decay is as follows:

$$\sum_{k=0}^{k(i)} |X_i(k)| = \frac{90}{100}\sum_{k=0}^{N-1} |X_i(k)|$$

Here, $|X_i(k)|$ represents the magnitude of the Fourier transform coefficient at frequency k for the ith frame.

9. Mel frequency cepstral coefficients(MFCCs)

Assuming p represents the Mel scale, these feature vectors are obtained under the condition of the first p Discrete Cosine Transform coefficients. In this study, the Mel scale (p) is set to 13, with the 13 extracted features denoted as $mfcc_1$ to $mfcc_{13}$.

10. Chroma vector

A vector containing 12 parameters ($chroma_1$ to $chroma_{12}$) represents the spectral energy at 12 pitch points within a segment. The standard deviation of these 12 parameters can be calculated and also used as an audio feature.
